# Locating the Acupoint Baihui (GV20) Beneath the Cerebral Cortex with MRI Reconstructed 3D Neuroimages

**DOI:** 10.1093/ecam/neq047

**Published:** 2011-02-14

**Authors:** Ein-Yiao Shen, Fun-Jou Chen, Yun-Yin Chen, Ming-Fan Lin

**Affiliations:** ^1^Graduate Institute of Acupuncture Science, China Medical University, Taichung, 40402, Taiwan; ^2^Department of Pediatrics, Taipei Branch, China Medical University Hospital, Taipei, 40402, Taiwan; ^3^Graduate Institute of Integrated Medicine, China Medical University, Taichung, 40402, Taiwan; ^4^Department of Radiology, Taipei Medical University-Wan Fang Hospital, Taipei, Taiwan, Taiwan

## Abstract

Baihui (GV20) is one of the most important acupoints of the Du meridian (the government vessel) and is commonly used in neurology and psychiatry and as a distal point of anorectal disorders by general practitioners. The anatomical relationship between the scalp region of the acupoint and the underlying corresponding cortex remains obscure. In this study, we first prepared the indicator for MRI scanning on a GE 1.5 T excite machine in a mode suitable for 3D reconstruction. The 3D Avizo software system (version 6.0, Mercury Computer Systems, Inc., Germany) was then used for image processing and the resulting data subsequently analyzed using descriptive statistics and analysis of variance (ANOVA). The mean distance from the Baihui anterior to the central sulcus in the adult group was greater than that in the child group (22.7 ± 2.2 and 19.7 ± 2.2 mm, resp., *P* = .042), whereas in the child group the distance between the Baihui anterior and the precentral sulcus was greater than in the adult group (6.8 ± 0.8 and 3.8 ± 0.8 mm, resp., *P* < .001). This MRI presentation demonstrates that the location of Baihui (GV20) can be identified using the distance from the central or precentral sulcus.

## 1. Introduction

Acupuncture therapy is a well-accepted therapeutic regimen in Chinese society. Scalp acupuncture can attenuate cerebral ischemia-reperfusion injury, improve neurofunctional rehabilitation and suppress leukocyte infiltration in rats [1, 2]. According to traditional Chinese medicine (TCM), acupoints are distributed along the meridians beneath the body's surface [3]. There are 14 meridians (channels) altogether, including 12 classical meridians—three on each side of the ventral part of the limbs and three on each side of the dorsal aspect; the other two meridians, the Ren meridian on the midline of the ventral part and the Du meridian (government vessel) on the midline of the dorsal aspect, are located on the midline of the human body. Many researchers have investigated these acupoints from a Western medical viewpoint using an anatomical approach, and numerous methodologies including X-rays [4], computer tomography (CT) scans [5] and autopsies [6] have been used to demonstrate the anatomy of the acupoints as mapped onto the human organs. These studies may be useful for identifying the focal therapeutic effect of these points. In recent years, as software has developed, we have become able to reconstruct human organs in a 3D image from 2D images of conventional CT or magnetic resonance imaging (MRI) slices, which has made it possible to study acupoints utilizing technology found in Western medicine. Baihui (GV 20) is a point on the Governor Vessel, and it is located on the highest place of the head where all the yang meridians meet [7–9], and the main therapeutic effects of GV20 are usually relief of headache, stroke, dizziness, and anxiety [10, 11]. The frontal lobes are involved in motor function, problem-solving, spontaneity, memory, language, initiation, judgment, impulse control, and social and sexual behavior. The parietal lobe plays an important role in integrating sensory information from various parts of the body, knowledge of numbers and their relationships, and in the manipulation of objects. If the acupoint Bauhui is related to the area beneath the cerebral cortex, we hypothesize that GV20 should be located in the frontal lobe area. This study presents our experience in localizing the acupoint Baihui (GV20) to the area beneath the cerebral cortex using reconstructed 3D neuroimages.

## 2. Methods

### 2.1. Subjects

From January 2004 to June 2007, 12 patients including 6 adults and 6 children underwent 3D neuroimaging analysis of the brain following receipt of consent from the patients themselves or from parents in the cases of children. The hospital's Ethics Committee also approved the study protocol.

## 3. Procedure

We began by setting up the indicator for MRI scanning on a GE 1.5 T excite machine in a mode suitable for 3D reconstruction. The brain of each patient was then scanned in transverse planes of 1.5 mm or less in order to obtain at least 80–120 slices, and the scans collated for algorithmic reconstruction and transmitted from the magnetic resonance unit database to an established workstation, whereupon the 3D Avizo software system (version 6.0, Mercury Computer Systems, Inc., Germany) was used for image processing. Technological advances in digital image processing using algorithms make it possible to practically, clearly, effectively and efficiently construct 3D images. Using this 3D reconstruction procedure, we can display the entire head and identify the location of acupuncture points on the brain using 3D images that include the ears, nose and glabella.

The image files were imported into the 3D Avizo software for image segmentation, and regions of interest were identified by the software's “brush" and “magic wand" functions. Grayscale values were limited to the scale range in order to approximate the boundary of the cortex (e.g., 75–95 of 1024 scales). The skull component of the brain was visually removed from the regions of interest by using an arithmetic module that isolated the cortex component. We then used the “voltex" function to display the whole head, including the ears, nose and glabella. This module provides direct volume rendering, which proved to be a very intuitive method for visualizing 3D scalar fields. Each point in a volume of data was assumed to emit and absorb light. The amount and the color of emitted light and the amount of absorption was determined from scalar data, which were derived from a color map with alpha values.

**Figure 1:**
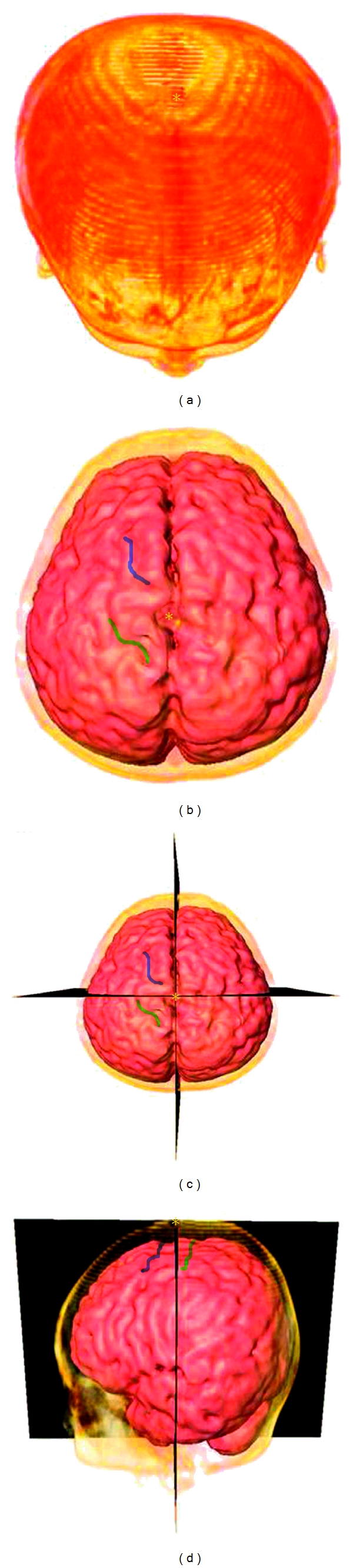
3D reconstructed image of the skull and cerebral cortex. (a) Vertical view of the cranium. (b) Vertical view of the 3D reconstructed cortical image and GV20 acupoint. (c) Mid-sagittal plane and ear lobe connecting coronal plane to demonstrate GV20 acupoint. (d) Lateral view with crossing of mid-sagittal plane and ear-lobe connecting coronal plane (blue region: precentral sulcus, green region: central sulcus, asterisk: Baihui acupoint).

There are four methods of acupoint location: two traditional methods (directional and proportional) and two contemporary methods (elastic and ruler) [12]. In order to evaluate the Baihui acupoint location, we needed to normalize and calibrate the MRI image for each patient. Both the gray scale intensity and the spatial orientation of the brain were important for our evaluation. We used the obliqueslice function by selecting the center-line from the top of the two ears with the glabella as the center and identified an upward straight line with crossover-point landmarks that help define the location of the Baihui acupoint (Figure 1). The obliqueslice module can display arbitrarily-oriented slices through a 3D scalar field of any type, as well as through an RGBA color field or a 3D multi-channel field. We sliced the sagittal plane along the local *x*-axis and the coronal plane along the local *y*-axis using the obliqueslice function in order to define the location of the Baihui acupoint. The distance between the Baihui acupoint and the central sulcus of each patient was measured from the left lateral-view image. 

### 3.1. Statistical Analysis

All data were analyzed using the SPSS10.0 statistical package. The distances from Baihui (GV20) to the central and precentral sulcus in adults and children were analyzed using descriptive statistics and ANOVA. All *P*-values were two-tailed, and the significance value was set at .05.

## 4. Results

### 4.1. Samples Characteristics

Twelve subjects had complete brain MRI image data portfolios and were analyzed. Three elderly males and three elderly females of normal intelligence and normal mental function were selected to form the adult group, the average age of which was 54.8 ± 14.0 years. Four boys and two girls with normal brain development were chosen to form the child group, of an average age of 6.2 ± 3.2 years.

### 4.2. 3D Reconstructed Image of the Cerebral Cortex

Anatomical analyses of GV20 and images of crossing of the sagittal and coronal planes are shown in Figure 1. We created an individual three-layer (scalp, skull and brain) head model from each subject's MRI scan. Although the brain image model has a great potential for human brain mapping, it seems to be difficult to use in routine experiments and clinical examinations, possible due to the one factor. It is not always possible to use MRI (slice from brainstem to vertex) to construct a subject's own head model [13].

### 4.3. Comparisons between the Adult and Child Groups

**Figure 2:**
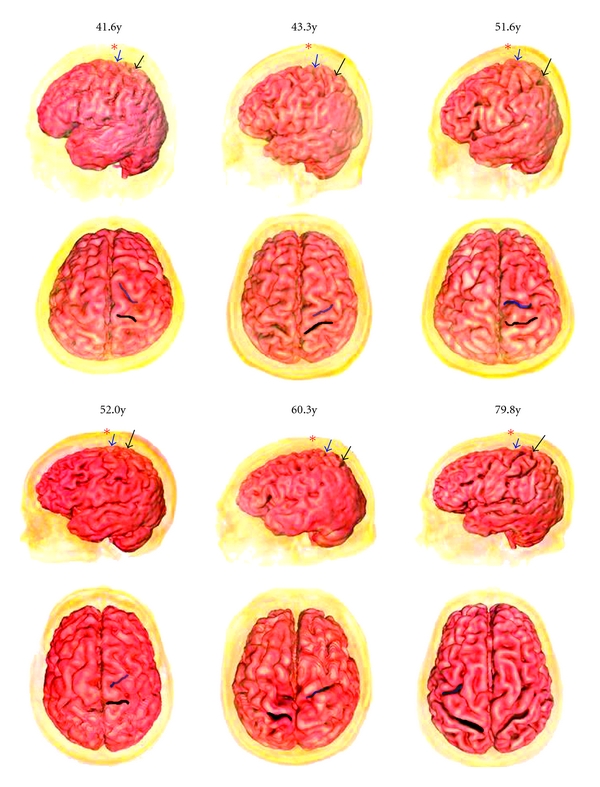
Left lateral view and planform of 3D reconstructed image of the cerebral cortex of six adults (asterisk: GV20 acupoint, blue arrow and region: precentral sulcus, black arrow and region: central sulcus).

**Figure 3:**
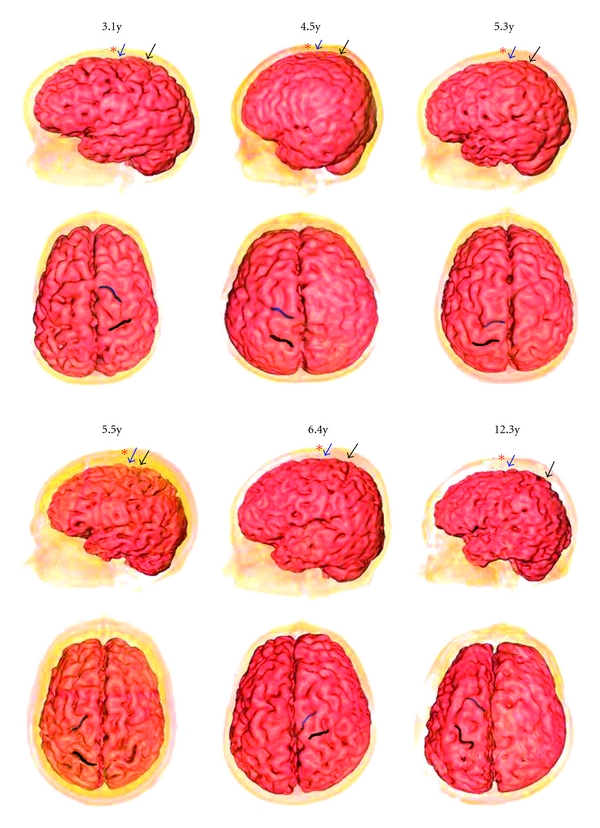
Left lateral view and planform of 3D reconstructed image of the cerebral cortex of six children (asterisk: GV20 acupoint, blue arrow and region: precentral sulcus, black arrow and region: central sulcus).

**Figure 4:**
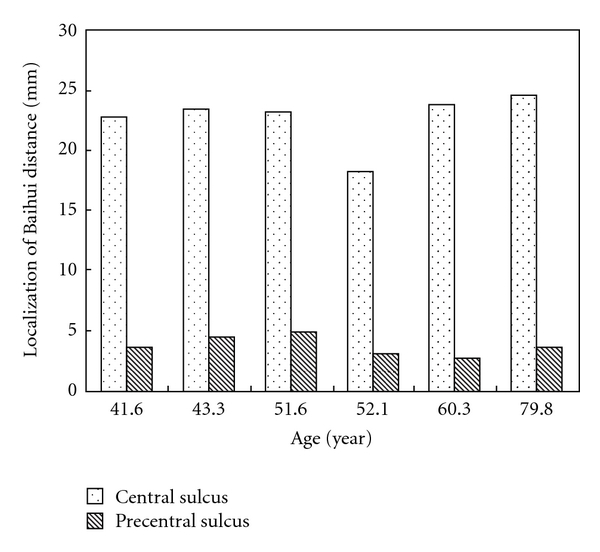
Localization of Baihui (GV20) to the central and precentral sulcus in adults.

**Figure 5:**
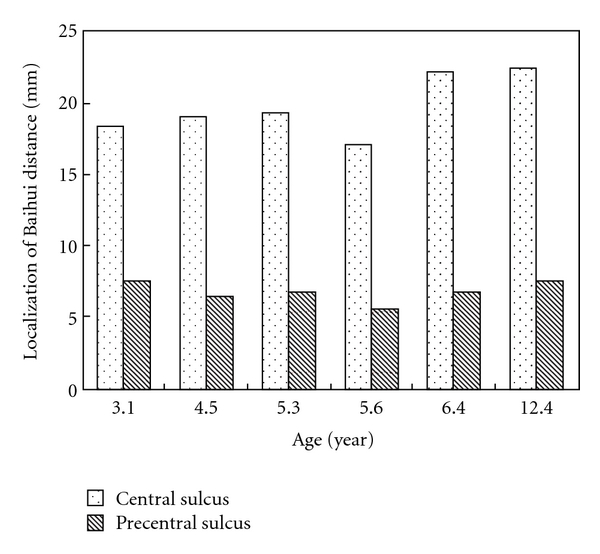
Localization of Baihui (GV20) to the central and precentral sulcus in children.

Twelve subjects related to localization of the central sulcus and precentral sulcus were identified. Differences between the adult group and the child group with respect to the position of the Baihui point as measured from the distance between the anterior and the central sulcus and precentral sulcus were found (Figures 2 and 3). Obviously, the mean distance from the Baihui anterior to the central sulcus in the adult group was greater than that in the child group (22.7 ± 2.2 and 19.7 ± 2.2 mm, resp.). Figures 4 and 5 show that there were significant differences found as *P* = .042; furthermore, a difference between groups was also observed in the distance between the Baihui anterior and the precentral sulcus, that in the child group being greater than that in the adult group (6.8 ± 0.8 and 3.8 ± 0.8 mm, resp.), the *P*-value for which was .001. 

## 5. Discussion

3D image processing technology is commonly used in scientific research [14], as it has been in movies, video games and industrial applications in the last two decades. The development of digital image processing makes it possible to display a 3D image practically, effectively and efficiently [15]. We employed a high-resolution grayscale method to define each part of the brain, skull and extracranial tissue in this study, and were therefore able to construct images of the scalp, skull and cerebral cortex, as well as the organs of the head such as the eyes and ear lobes, and the anatomy of each part of the intracranial and extra cranial organ tissues.

Baihui (GV20) is one of the most important acupoints of the government vessels and of the entire meridian acupoint system. It is responsible for enhancing nitric oxide (NO) generation and increasing local circulation; it is also used for treating conditions such as headache, stroke, dizziness, and anxiety [16, 17]. This point has a stimulatory effect on the central nervous system rather than just a peripheral nerve; we believe it stimulates the central nervous system (the brain and spinal cord) to release neurotransmitters or neuromodulators into the brain and spinal cord, chemicals that either change the experience of pain or release other chemicals, such as hormones, that influence the body's self-regulation systems. These biochemical changes may stimulate the body's natural healing abilities and promote physical and emotional well-being. There are three main mechanisms of biochemical change: conduction of electromagnetic signals, activation of opioid systems, and changes in brain chemistry, sensation and involuntary body functions.

The location of Baihui (GV20) is easy to define according to TCM and the definition of the WHO [18]. Ordinarily, it is said to be located at the intersection of the line connecting the lowest and highest points of the ear lobe and the median line of the head, 7 cun above the posterior hairline and 5 cun behind the anterior hairline. This study defines the location of GV20 as the intersection point of the midline and the line connecting the ear lobes as calculated from the 3D images of the head (Figure 1) [19].

The reason for Baihui (GV20) needling is predominantly to treat psychological maladies, as there are general sedative and harmonizing effects. This point is ordinarily used in every acupuncture treatment because of its general psychological effects; it may also be effective for treating insomnia, anxiety, headaches, apoplexy, and weakness of memory. The frontal lobe is mainly responsible for these disorders or symptoms [20, 21].

It was found in this study that the location of the Baihui (GV20) acupoint is in the scalp, 22.7 ± 2.2 mm anterior to the central sulcus and 3.8 ± 0.8 mm anterior to the precentral sulcus in adults, and 19.7 ± 2.2 mm anterior to the central sulcus and 6.8 ± 0.8 mm anterior to the precentral sulcus in children. These data may not be an absolutely accurate description of the location due to the limitations of case numbers and the image resolution power of the machine used. However, this study demonstrates that the GV20 is located in the area of the frontal lobe anterior precentral sulcus.

The functional correlation between the stimulated point and the corresponding underlying cortex requires further study. Functional-anatomical imaging methods such as PET-CT scans or functional MRI may be useful in demonstrating the immediate and late responses of the underlying cortex as well as the true functional area of the acupoint, and we are looking forward to performing such further research.

## Funding

China Medical University Hospital (DMR-99-040).
